# HIPPO Pathway Members Restrict SOX2 to the Inner Cell Mass Where It Promotes ICM Fates in the Mouse Blastocyst

**DOI:** 10.1371/journal.pgen.1004618

**Published:** 2014-10-23

**Authors:** Eryn Wicklow, Stephanie Blij, Tristan Frum, Yoshikazu Hirate, Richard A. Lang, Hiroshi Sasaki, Amy Ralston

**Affiliations:** 1 Molecular, Cell, and Developmental Biology, University of California Santa Cruz, Santa Cruz, California, United States of America; 2 Institute of Molecular Embryology and Genetics, Kumamoto University, Chuo-ku, Kumamoto, Japan; 3 Division of Pediatric Ophthalmology, Cincinnati Children's Hospital Medical Center, Cincinnati, Ohio, United States of America; University of Wisconsin, United States of America

## Abstract

Pluripotent epiblast (EPI) cells, present in the inner cell mass (ICM) of the mouse blastocyst, are progenitors of both embryonic stem (ES) cells and the fetus. Discovering how pluripotency genes regulate cell fate decisions in the blastocyst provides a valuable way to understand how pluripotency is normally established. EPI cells are specified by two consecutive cell fate decisions. The first decision segregates ICM from trophectoderm (TE), an extraembryonic cell type. The second decision subdivides ICM into EPI and primitive endoderm (PE), another extraembryonic cell type. Here, we investigate the roles and regulation of the pluripotency gene *Sox2* during blastocyst formation. First, we investigate the regulation of *Sox2* patterning and show that SOX2 is restricted to ICM progenitors prior to blastocyst formation by members of the HIPPO pathway, independent of CDX2, the TE transcription factor that restricts *Oct4* and *Nanog* to the ICM. Second, we investigate the requirement for *Sox2* in cell fate specification during blastocyst formation. We show that neither maternal (M) nor zygotic (Z) *Sox2* is required for blastocyst formation, nor for initial expression of the pluripotency genes *Oct4* or *Nanog* in the ICM. Rather, Z *Sox2* initially promotes development of the primitive endoderm (PE) non cell-autonomously via FGF4, and then later maintains expression of pluripotency genes in the ICM. The significance of these observations is that 1) ICM and TE genes are spatially patterned in parallel prior to blastocyst formation and 2) both the roles and regulation of *Sox2* in the blastocyst are unique compared to other pluripotency factors such as *Oct4* or *Nanog*.

## Author Summary

Pluripotent stem cells can give rise to any cell type in the body, making them an attractive tool for regenerative medicine. Pluripotent stem cells can be derived from the mammalian embryo at the blastocyst stage or they can be created from mature adult cells by reprogramming. During reprogramming, SOX2 helps establish pluripotency, but it is not clear how SOX2 establishes pluripotency in the blastocyst. We evaluated where SOX2 is present, how SOX2 is regulated, and where SOX2 is active during blastocyst formation. Our data show that the roles and the regulation of SOX2 are unique compared to other pluripotency/reprogramming factors, such as OCT4 and NANOG. SOX2 marks pluripotent cells earlier than do other factors, but does not regulate pluripotency until several days later. Rather, the earlier role of SOX2 is to help establish the yolk sac lineage, which is essential for gestation.

## Introduction

To create and use pluripotent stem cells, it is essential to understand the origins of pluripotency during normal development. During mouse blastocyst formation, pluripotent epiblast (EPI) cells are established by two cell fate decisions that segregate pluripotent progenitors from extraembryonic tissues [1], [2]. During the first cell fate decision, trophectoderm (TE) is segregated from inner cell mass (ICM) prior to blastocyst formation. During the second cell fate decision, the ICM is subdivided into EPI and primitive endoderm (PE) lineages after blastocyst formation. Recent studies have examined the roles and regulation of pluripotency genes, such as *Oct4*, *Nanog*, and *Sox2*, during establishment of EPI cells in the blastocyst [3]–[12], but aspects of the roles and regulation of *Sox2* in the blastocyst are unresolved. For example, several studies reported that *Sox2* is restricted to the ICM by the blastocyst stage [3], [13]–[15], but the molecular mechanisms regulating *Sox2* expression in the blastocyst are unknown.

In addition to the unresolved mechanism by which *Sox2* expression is patterned, the functional roles of *Sox2* in the blastocyst are not yet clear. ES cells cannot be derived from embryos lacking zygotic (Z) *Sox2* [5], indicating that *Sox2* is essential for pluripotency. In ES cells, *Sox2* is required for the expression of pluripotency genes, such as *Oct4* and *Nanog*, and for the repression of TE genes [16]–[20]. Therefore, *Sox2* might be required for initial expression of pluripotency genes and repression of TE genes in the ICM. However, the expression of pluripotency and TE genes in *Sox2* Z null blastocysts has not yet been examined at the level of individual cells. Moreover, maternal (M) *Sox2* is also thought to participate in blastocyst formation, which could partially compensate for loss of Z *Sox2*. RNAi knockdown of M and Z *Sox2* in the zygote was reported to disrupt blastocyst formation [6]. However, RNAi knockdown embryos do not always phenocopy MZ null embryos [3], [21]. Because understanding the regulation and roles of SOX2 in the blastocyst is key to understanding the molecular regulation of preimplantation development and the establishment of pluripotency, we examined both the mechanisms that pattern SOX2, as well as the functional requirements for MZ *Sox2* during development.

## Results

### SOX2 is restricted to ICM progenitors by HIPPO pathway members, and not by CDX2

**Figure 1:**
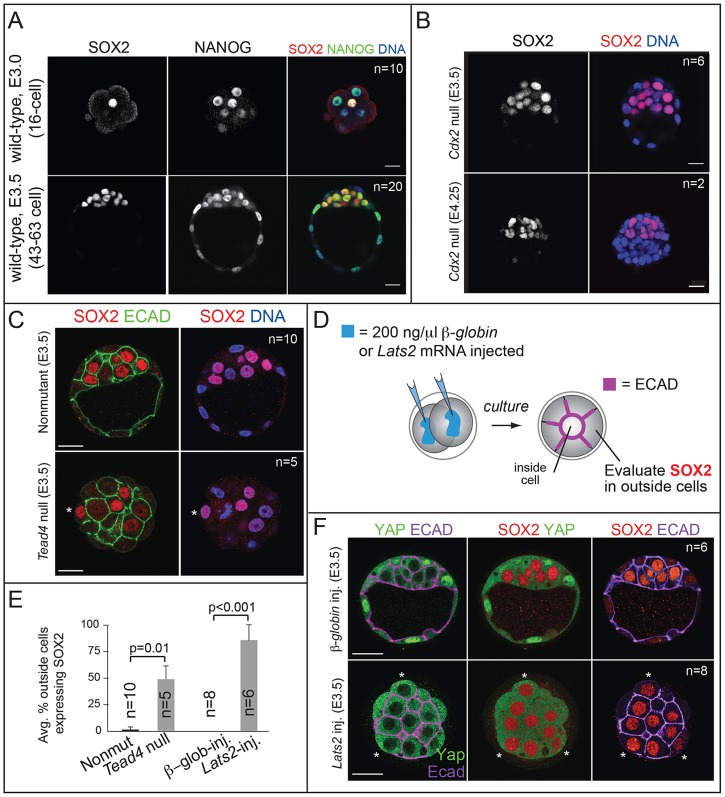
SOX2 is restricted to ICM progenitors by HIPPO pathway members and not by CDX2. A) Immunofluorescent analysis of SOX2 and NANOG shows that SOX2 is detected specifically in ICM cells at the 16-cell stage and later, while NANOG is detected in all cells at these stages. B) SOX2 is not upregulated in the TE of *Cdx2* null embryos at early or late blastocyst stages, indicating that CDX2 does not restrict SOX2 to the ICM. C) SOX2 is ectopically expressed in outside cells of embryos lacking the HIPPO pathway member *Tead4* (asterisk  =  SOX2-positive outside cell). TE cells are defined both by outside position and by basolateral localization of E-CADHERIN (ECAD). D) Either *Lats2* or *β-Globin* mRNAs were injected into both cells of 2-cell embryos, and embryos were then cultured to blastocyst stage. E) The proportion of outside cells in which SOX2 was ectopically expressed was significantly increased in both *Tead4* null embryos, and in embryos overexpressing the HIPPO pathway member *Lats2*, relative to controls (p-value calculated by t-test). F) Overexpression of *Lats2*, which prevents nuclear YAP localization, causes ectopic expression of SOX2 in outside cells (indicated by asterisk). In all panels, bar  = 20 µm.

**Figure 2:**
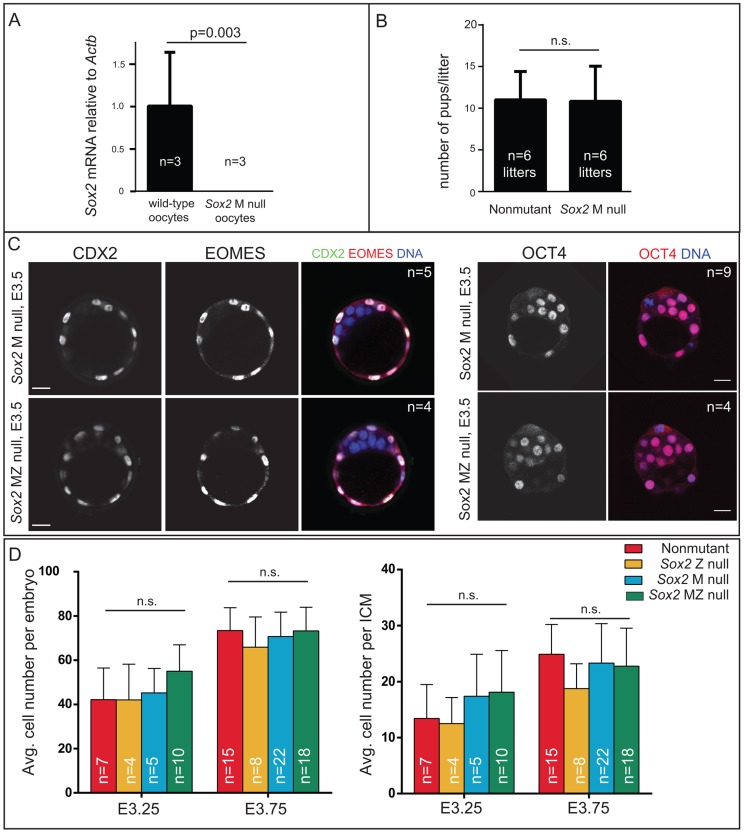
*Sox2* is not required for the first lineage decision: segregation of ICM and TE cell types. A) qPCR analysis confirms that *Sox2* is deleted in oocytes from females carrying *Zp3Cre* and the floxed *Sox2* allele (bars represent standard deviation from the average of 3 replicate pools of ∼10 oocytes each). B) M *Sox2* is not required for development because litter sizes did not significantly differ between non-mutant females, and females in which *Sox2* had been deleted in the germ line. C) The expression patterns of the TE markers CDX2 and EOMES and the ICM marker OCT4 are normal in embryos lacking *Sox2*. D) The number of total cells, inside (ICM) cells, and outside (TE) cells is normal in the absence of M, Z, or MZ *Sox2*. Bar  = 20 µm, p-value calculated by t-test in A, B; ANOVA performed in D; n.s.  = p>0.05.

*Sox2* mRNA is enriched in ICM progenitors starting at the 16-cell stage [14], but the SOX2 protein expression pattern at this stage is unclear, as is the mechanism by which *Sox2* is restricted to ICM progenitors. Using immunofluorescence and confocal microscopy, we observed that SOX2 is restricted to nuclei of ICM progenitors at the 16-cell stage and later (Fig. 1A; see Table S1 for wild-type embryo staging scheme). In morulae, a weaker signal was detected in the cytoplasm of outside cells, but this was also detected in embryos lacking MZ *Sox2* (Fig. S1A), indicating that the cytoplasmic stain is non-specific. In the early blastocyst (E3.25–E3.5), SOX2 was detected in most ICM cells (Fig. 1A and Fig. S1B), and SOX2 did not colocalize with CDX2 in outside cells (n = 13 embryos; Fig. S1C). By contrast, NANOG and OCT4 are still detected in the TE at this stage (Fig. 1A and 2C) [22], [23]. Therefore SOX2 is a unique, early marker of ICM fate.

Next we examined the mechanism by which SOX2 expression is restricted to ICM. The TE-expressed transcription factor CDX2 restricts the expression of *Oct4* and *Nanog* to the ICM by repressing *Oct4* and *Nanog* expression in the TE after blastocyst formation [22]. We therefore asked whether CDX2 also restricts SOX2 to the ICM. Surprisingly, SOX2 remained restricted to the ICM in *Cdx2* null embryos at early and late blastocyst stages (Fig. 1B), indicating that SOX2 expression is restricted to ICM progenitors through a *Cdx2*-independent mechanism. We therefore investigated whether the pathway that restricts CDX2 to the TE also restricts SOX2 to the ICM in parallel. We previously helped show that TEAD4 partners with YAP and WWTR1 to promote expression of CDX2 and GATA3 in TE cells [7], [24]–[26]. YAP and WWTR1 are localized to nuclei only in TE cells, where LATS kinase activity is lower [26]. We hypothesized that if TEAD4 regulates *Sox2* in parallel to *Cdx2*, then we would detect ectopic SOX2 in the TE cells of *Tead4* null embryos. To test this hypothesis, we examined SOX2 expression in *Tead4* null embryos. *Tead4* is essential for blastocyst formation, but not for polarization of TE cells [24], [25], enabling us to distinguish inside (apolar) and outside (polarized) cells in *Tead4* null embryos. We observed that in *Tead4* null embryos, ectopic SOX2 was detected in about half of outside cells, in contrast with control littermates, where SOX2 was not detected in outside cells (Fig. 1C, E). Thus, *Tead4* is required to restrict SOX2 to ICM cells. This result is significant because although other pluripotency factors, such as OCT4 and NANOG are detected in outside cells of *Tead4* null embryos when they die [24], [25], these factors are also detected in the outside cells of wild type embryos at this stage [22]. SOX2 is therefore the first pluripotency factor known to be restricted to ICM progenitors by TEAD4 and not by CDX2.

Finally, we misexpressed *Lats2* mRNA in outside cells (Fig. 1D), which is sufficient to shift YAP and WWTR1 localization from nucleus to cytoplasm and downregulate CDX2 in TE cells [26]. As a control, we overexpressed β-*Globin* in a second group of embryos. As expected, overexpression of *Lats2* disrupted nuclear YAP localization and led to ectopic SOX2 in outside cells, while ectopic of β-*Globin* did not (Fig. 1E, F). Therefore, LATS2 and TEAD4 regulate patterning of SOX2 and CDX2 in parallel, leading to the establishment of their complementary expression patterns in the blastocyst.

### Maternal *Sox2* is not required for development

Our finding that SOX2 protein is restricted to ICM progenitors conflicted with prior reports suggesting that M SOX2 protein is present in TE cells and required for TE cell development [5], [6]. However, the requirement for MZ SOX2 in the TE has not been functionally evaluated using null alleles. We examined embryos lacking MZ *Sox2* using a conditional *Sox2* allele [27] and *Zp3Cre*, which is expressed in the female germ line [28]. We first confirmed that female germ line expression of *Cre* indeed deleted M *Sox2* by quantitative RT-PCR (qPCR) analysis of oocytes from *Zp3Cre; Sox2^fl/fl or del^* females (Fig. 2A). Next, we mated these females to wild-type males to determine whether M *Sox2* is required for development of their progeny. The number of offspring per litter did not differ significantly between *Sox2* germ line-deleted females and control females (Fig. 2B), indicating that M *Sox2* is not required for development, consistent with a recent report [29]. While variable levels of *Sox2* mRNA have been detected in 1–2 cell embryos [14], we were unable to detect *Sox2* mRNA or protein in wild type embryos at 1–2 cell stages, or in *Sox2* Z null morulae (Fig. S2A), indicating that M SOX2 is neither present nor functional in the blastocyst.

We next examined whether loss of both M and Z *Sox2* disrupts blastocyst formation by breeding *Sox2* germ line-deleted females to males carrying a *Sox2* null allele. Expression of TE (CDX2 and EOMES) and ICM (OCT4) markers was normal in the absence of MZ *Sox2* (Fig. 2C), indicating that neither M nor Z *Sox2* is required for TE specification or blastocyst formation, in contrast with the reported RNAi phenotype [6]. Moreover, quantification of the numbers of ICM and total cells in the blastocyst showed that there was no significant reduction in the average numbers of cells contributing to either lineage in the absence of MZ *Sox2*, compared to other genotypes (Fig. 2D). In ES cells, deletion of *Sox2* leads to downregulation of *Oct4* and upregulation of TE genes, including *Eomes* [16]. However, we did not detect ectopic expression of TE genes in ICM cells of *Sox2* MZ null blastocysts at early (Fig. 2C) or late (Fig. S2B) time points. We therefore conclude that neither M nor Z *Sox2* are required for segregation of ICM and TE lineages.

### In the ICM, SOX2 is restricted to EPI cells by an FGF4/MEK-dependent mechanism

Our results indicated that *Sox2* is not required for the first lineage decision, so we next asked if *Sox2* is involved in the second lineage decision during mouse development: the subdivision of the ICM into EPI and PE cell fates. In the blastocyst at E3.75, EPI and PE cells are distributed in a salt-and-pepper fashion within the ICM. EPI cells express higher levels of NANOG, while PE cells express higher levels of SOX17, GATA6, PDGFRA, and GATA4 [4], [30]–[32]. In contrast, OCT4 is detected in both EPI and PE cells at this stage [4], [14], [23], [33]. It is not currently known whether SOX2 is restricted to EPI cells like NANOG, or if SOX2 is expressed throughout the ICM like OCT4. We therefore first examined the SOX2 pattern within the ICM in the E3.75 blastocyst.

**Figure 3:**
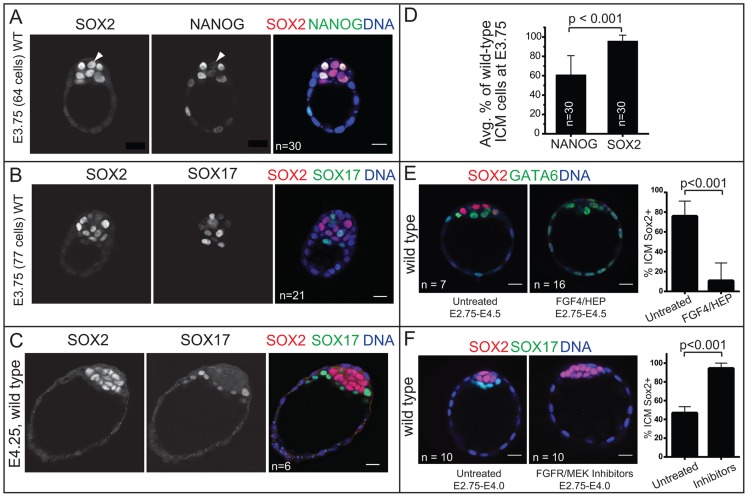
*Sox2* is restricted to EPI progenitors through an Fgf4/MEK-dependent mechanism. A) In wild-type (WT) embryos at early E3.75, NANOG is detected in a salt-and-pepper pattern in the ICM, while SOX2 begins to be downregulated in PE cells (arrowhead: cell in which NANOG is already downregulated, but SOX2 is not yet downregulated). B) In WT embryos at late E3.75, SOX2 and SOX17 are detected in a salt-and-pepper pattern in the ICM. C) At E4.25, SOX2 is exclusively detected in EPI and SOX17 in PE. D) At E3.75, SOX2 is detected in a larger proportion of ICM cells than is NANOG, indicating that NANOG is downregulated in the PE slightly before SOX2. E) FGF4/HEP is sufficient to repress SOX2 expression in the ICM since the SOX2-expressing proportion of ICM cells is reduced (and GATA6-expressing proportion concomitantly expanded) in wild-type embryos incubated in FGF4/HEP (avg. no. untreated ICM cells: 25.6+/−3.8; avg. no. treated ICM cells: 30.4+/−7.2). F) The downregulation of SOX2 in PE cells is dependent on FGFR/MEK, since the proportion of ICM cells expressing SOX2 is expanded (and the SOX17-expressing proportion concomitantly reduced) in wild-type embryos incubated in inhibitors of FGFR/MEK (avg. no. untreated ICM cells: 19.4+/−5.1; avg. no. treated ICM cells: 13+/−4.1). Bar  = 20 µm, p-value calculated by t-test.

Our analysis of early and late blastocysts indicated that the SOX2 expression pattern in the ICM resembles that of NANOG and not OCT4. At E3.75, SOX2 was detected in cells expressing NANOG and not in cells expressing SOX17 (Fig. 3A, B), although downregulation of NANOG in PE cells slightly preceded the downregulation of SOX2 in the PE cells (Fig. 3A, D). By E4.25, SOX2 was detected in EPI and not PE cells (Fig. 3C). These observations suggested that SOX2 and NANOG are restricted to EPI cells by a similar mechanism. NANOG has been shown to be restricted to EPI cells by FGF4/MEK [9], [34]–[37], so we evaluated whether FGF/MEK signaling is necessary and/or sufficient to repress SOX2 in the ICM. We cultured wild-type embryos in FGF4 and HEPARIN (FGF4/HEP) from E2.75 to E4.5, which leads to repression of NANOG, and upregulation of SOX17 in the ICM [3], [34]. Control embryos were cultured alongside in the absence of FGF4/HEP. We observed that FGF4/HEP was sufficient to repress SOX2 and upregulate SOX17 in wild-type embryos (Fig. 3E). Next, we cultured wild-type embryos in inhibitors of FGFR/MEK from E2.75 to E4.0, which leads to ectopic expression of NANOG and repression of SOX17 in the ICM of wild-type embryos [34], [36]. Treatment with FGFR/MEK inhibitors led to ectopic expression of SOX2 and repression of SOX17 throughout the ICM (Fig. 3F). We conclude that SOX2 expression is restricted to EPI cells through an FGFR/MEK-dependent mechanism.

### *Sox2* is required for initial expression of PE genes

**Figure 4:**
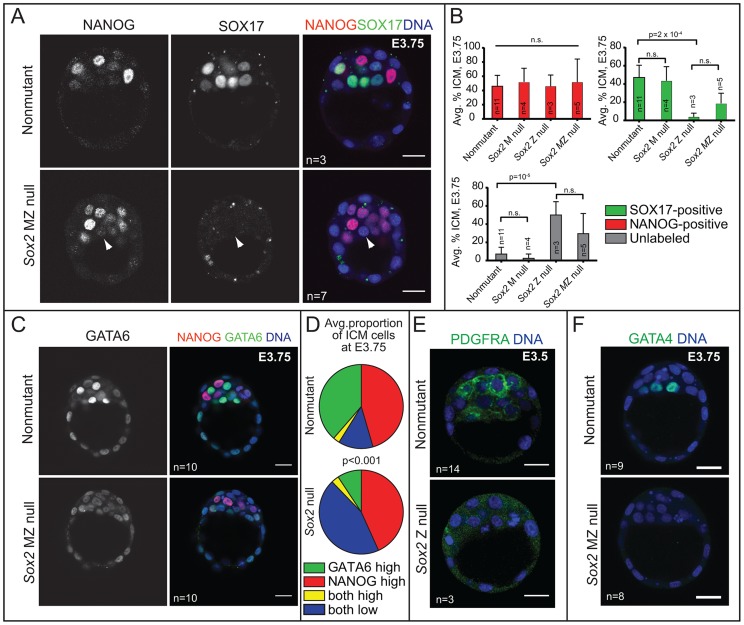
*Sox2* is required for the initial expression of PE genes in the ICM. A) At E3.75, NANOG is detected in *Sox2* null embryos, but SOX17 is not detected in most *Sox2* null embryos (arrowhead  =  ICM cell expressing neither NANOG nor SOX17). B) At E3.75, the average proportion of ICM cells in which NANOG is elevated is equivalent among all genotypes examined, indicating that *Sox2* is not required for expression of NANOG in the ICM. However, the average proportion of ICM cells in which SOX17 is detected is significantly reduced, and the proportion of ICM cells in which neither NANOG nor SOX17 are detected is significantly increased, in the absence of either Z or MZ *Sox2*. C) At E3.75, *Sox2* is required for high levels of GATA6 in PE cells. D) Quantification of immunofluorescent results showing that the proportion of ICM cells in which high levels of GATA6 are detected is significantly lower *Sox2* null embryos at E3.75, while the proportion of ICM cells expressing both NANOG and low levels of GATA6 is significantly higher. E) Expression of PDGFRA in the ICM depends on *Sox2*. F) Expression of GATA4 in the ICM depends on *Sox2*. Bar  = 20 µm, p-value calculated by t-test in B, and by Chi-squared test in E; n.s.  = p>0.05.

Our results showed that, like NANOG, SOX2 expression is restricted to EPI cells at E3.75 and later. We next asked whether *Sox2* is required for the expression of NANOG or for the segregation of EPI and PE cell types at this stage. Prior analysis of *Sox2* Z null embryos showed that formation of the ICM is independent of Z *Sox2* [5], but the requirement for M *Sox2* was not evaluated. To determine whether MZ *Sox2* is required for NANOG expression, we evaluated whether the ICM contained normal numbers of NANOG-expressing cells in the absence of MZ *Sox2*. NANOG was detected at normal levels in the absence of MZ *Sox2* at E3.75 (Fig. 4A), and in a normal number of ICM cells in *Sox2* M, Z, and MZ null blastocysts at E3.75 (Fig. 4B). These observations indicate that *Sox2* is dispensable for regulating the initial expression of NANOG.

By contrast, the number of cells in which we detected the PE marker SOX17 was greatly reduced in *Sox2* null blastocysts relative to control embryos at E3.75. In these embryos, the number of unlabeled cells, in which neither NANOG nor SOX17 was detected, was greatly increased in the absence of *Sox2* (Fig. 4A, B). Notably, this phenotype was equivalent between *Sox2* Z and MZ null embryos (Fig. 4B), consistent with the conclusion that there is no role for M *Sox2*. We also examined other PE markers and found that the proportion of ICM cells expressing a higher level of GATA6 was significantly reduced in the absence of *Sox2* (Fig. 4C, D), and both PDGFRA and GATA4 were detected at much lower levels in the absence of *Sox2* (Fig. 4E, F). These results indicate that *Sox2* promotes PE gene expression at E3.5–E3.75.

### *Sox2* promotes PE gene expression via FGF4

**Figure 5:**
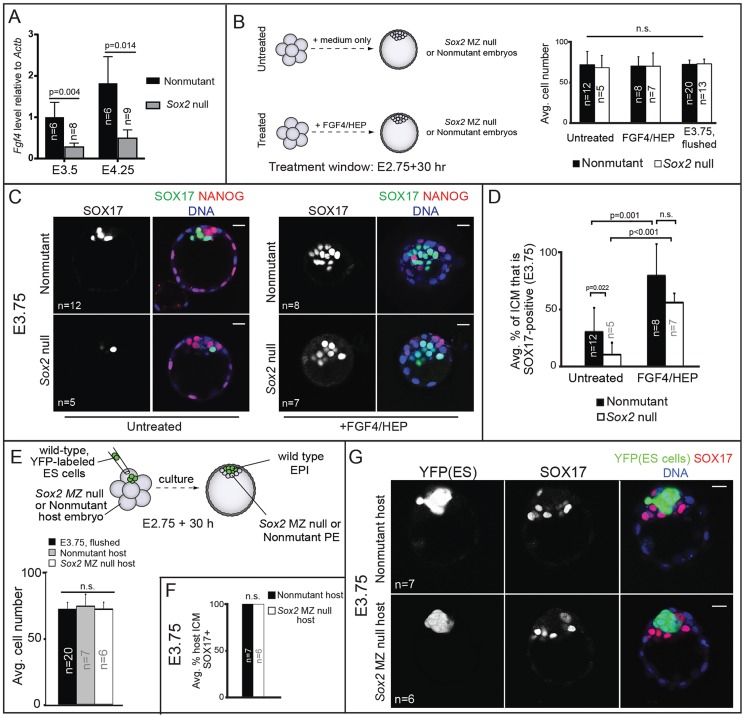
*Sox2* promotes PE development non cell-autonomously via FGF4. A) Expression of *Fgf4*, as measured by qPCR, is reduced in the absence of *Sox2*. B) Treatment scheme and evidence that resultant embryos are equivalent in cell number to developmental stage E3.75. C) FGF4/HEP treatment is sufficient to induce expression of SOX17 in *Sox2* null embryos. D) Quantification of the experiment shown in panel C. E) Overview of strategy to generate chimeric embryos and evidence that chimeras are equivalent in cell number to E3.75 (avg. no. host cells per ICM: untreated nonmutant: 19.1+/−6.2; untreated *Sox2* null: 20.0+/−4.1; treated nonmutant: 11.2+/−3.6; treated *Sox2* null: 12.0+/−3.2). F) Wild-type ES cells rescue expression of SOX17 in *Sox2* null embryos. G) Chimeras from panel F. Bar  = 20 µm, p-value calculated by t-test; ANOVA performed for panels B, D, E; n.s.  = p>0.05.

To discover the mechanism by which *Sox2* promotes PE development, we next examined the role of *Sox2* in regulating *Fgf4* expression, since *Fgf4* is necessary and sufficient to induce PE gene expression in the ICM [34], [35], [37]. SOX2, together with OCT4, promotes expression of *Fgf4* in pluripotent stem cell lines [38], [39], indicating that *Sox2* may also promote expression of *Fgf4* in the ICM. Consistent with this hypothesis, *Fgf4* mRNA was reduced in *Sox2* null blastocysts to about 30% of the wild-type level (Fig. 5A). Additionally, we found no requirement for M *Sox2* in promoting expression of *Fgf4* (Fig. S3A). Thus Z *Sox2* is required for maximal expression of *Fgf4* in the blastocyst, leading us to ask whether the observed defects in PE gene expression in *Sox2* Z null blastocysts are due to reduced expression of *Fgf4*.

If the disruption in PE gene expression in *Sox2* null embryos were due to reduced *Fgf4* expression, then exogenous FGF4/HEP should restore PE gene expression. To test this prediction, we cultured *Sox2* null embryos in FGF4/HEP from the 8-cell stage (E2.75) to E3.75 (Fig. 5B). As a positive control, we cultured non-mutant embryos in FGF4/HEP, and as a negative control we cultured *Sox2* null embryos in the absence of exogenous FGF4/HEP. We first confirmed that embryos of all genotypes and treatment groups were equivalent to the E3.75 developmental stage in terms of cell number (Fig. 5B). Next, we evaluated expression of SOX17 and NANOG in each group. As predicted, FGF4/HEP treatment led to a significant increase in the proportion of SOX17-positive ICM cells in *Sox2* null and non-mutant embryos, relative to untreated *Sox2* null and non-mutant embryos (Fig. 5C, D). We also cultured *Sox2* null and non-mutant embryos in FGF4/HEP for an extended period, after which 100% of ICM cells became SOX17-positive/NANOG-negative, irrespective of genotype (Fig. S3B, C), confirming that *Sox2* null embryos respond to exogenous FGF4/HEP like non-mutant embryos. We conclude that *Sox2* is not required for ICM cells to receive or respond to FGF4 signaling, but is required for maximal expression of *Fgf4*.

Our observations that *Sox2* promotes PE gene expression via FGF4 predicts that *Sox2* promotes PE gene expression non cell-autonomously. We tested this hypothesis by examining expression of PE genes in chimeric embryos containing a *Sox2* null PE and wild-type EPI. To generate these chimeras, we aggregated wild-type, YFP-expressing ES cells [40] with precompacted 8-cell *Sox2* null or non-mutant host embryos, and then cultured these chimeras to E3.75 (Fig. 5E). Performing the aggregation at this stage allows the ES cells to completely colonize the EPI compartment, such that only PE and TE cells are host-derived [3], [41]. We observed that in *Sox2* null host embryos, expression of SOX17 was rescued by wild type ES cells (Fig. 5F, G). These results indicate that *Sox2* in EPI cells acts non cell-autonomously to promote expression of SOX17 in PE cells by E3.75.

### *Sox2* maintains EPI, but not PE, gene expression

**Figure 6:**
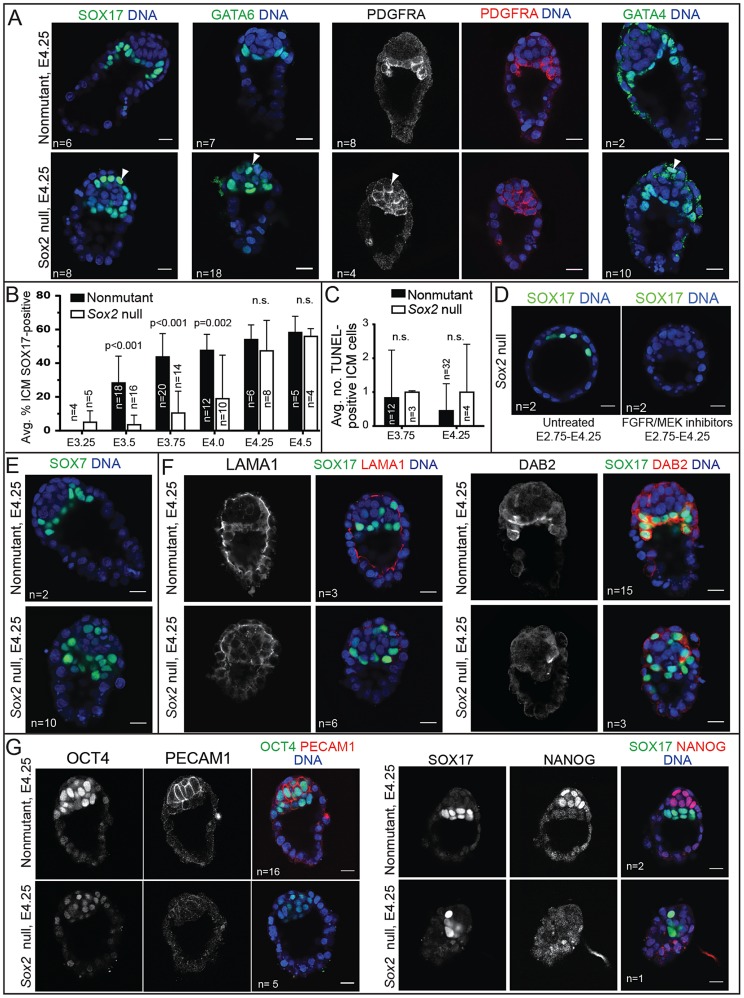
*Sox2* is required to maintain expression EPI, but not most PE genes. A) By E4.25, expression of PE genes, including SOX17, PDGFRA, and GATA4, is restored in *Sox2* null embryos, but the ICM appears disorganized relative to control embryos. (arrowheads  =  mislocalized PE cells. B) In *Sox2* null embryos, the average proportion of ICM cells expressing SOX17 increases progressively, catching up with control embryos by E4.25. C) Quantification of the average number of apoptotic cells in wild type and *Sox2* null embryos at the indicated time points. D) At E4.25, the expression of SOX17 in *Sox2* null embryos depends on FGFR/MEK. E) At E4.25, SOX7, a marker of mature PE, is detectable in the absence of *Sox2*. F) At E4.25, expression of LAMA1 and DAB2 are reduced in the absence of *Sox2*, consistent with defects in PE localization. G) At E4.25, expression of OCT4, PECAM1, and NANOG are reduced in the absence of *Sox2*. Bar  = 20 µm, p-value calculated by t-test; n.s.  = p>0.05.

We next examined whether *Sox2* is required to maintain the expression of PE genes after E3.75. Surprisingly, we detected SOX17, GATA6, PDGFRA, and GATA4 in *Sox2* null embryos at E4.25 (Fig. 6A). By examining the time course of SOX17 expression in *Sox2* null embryos, we determined that SOX17 was detected in a progressively larger proportion of ICM cells in *Sox2* null embryos starting from E3.25 until E4.25, when the proportion of SOX17-expressing cells was equivalent to wild type (Fig. 6B). Similarly, the proportion of cells expressing a high level of GATA6 was also normal in *Sox2* null embryos at E4.25 (Fig. S4A). These results show that *Sox2* is required for the initial, but not the later, expression of SOX17, GATA6, PDGFRA, and GATA4.

We hypothesized that PE gene expression is eventually induced in the cells that were originally unlabeled in *Sox2* null embryos at E3.75 (Fig. 4B). Alternatively, rare, correctly specified PE cells may have proliferated to replace the unlabeled cells in *Sox2* null embryos. This latter hypothesis predicts that unlabeled cells would undergo apoptosis in *Sox2* null embryos to maintain ICM cell number from E3.75 to E4.25 (Fig. S4B). However, we did not observe a difference in the number of apoptotic cells in *Sox2* null embryos during this window (Fig. 6C), suggesting that PE gene expression is eventually induced in cells that were originally unlabeled in *Sox2* null embryos at E3.75. We hypothesized that the delayed expression of PE genes in *Sox2* null embryos is due to the lower level of *Fgf4* (Fig. 5A). Consistent with this hypothesis, the expression of SOX17 in *Sox2* null embryos at E4.25 was indeed dependent on FGFR/MEK signaling (Fig. 6D), arguing that low levels of FGF4 can eventually induce expression of PE genes in *Sox2* null embryos. To determine whether delayed PE gene expression also delayed PE maturation, we examined expression of SOX7, which is expressed only in mature PE cells [42]. At E4.25, SOX7 was detected in *Sox2* null embryos (Fig. 6E), suggesting that PE cells had matured in an age-appropriate manner, in spite of the reduced *Fgf4*. We conclude that *Sox2* is not required for maintaining PE gene expression in the blastocyst, consistent with the observation that PE-derived cells are detected in *Sox2* null embryos postimplantation [5].

Curiously, we noted that in spite of the normal expression of PE genes in the absence of *Sox2*, PE cells were often mislocalized in *Sox2* null embryos at E4.25. Rather than forming a single, contiguous hypoblast layer between EPI and blastocoel, PE cells were often observed between EPI and polar TE in *Sox2* null embryos (Fig. 6A, E). These observations raised the possibility that *Sox2* promotes expression of genes thought to regulate PE cohesion and sorting, such as LAMININ and DAB2 [43]–[45], which are normally detectable in the PE at E4.25 [31], [42], [46]. Consistent with this hypothesis, expression of both LAMA1 and DAB2 was reduced in PE cells at E4.25 in *Sox2* null blastocysts (Fig. 6F). Notably, expression of DAB2 and PE sorting were rescued by wild type ES cells in *Sox2* null E4.25 blastocysts (Fig. S4C), and expression of DAB2 was eventually restored in implantation-delayed blastocysts (Fig. S4D), consistent with SOX2 acting non cell-autonomously to promote the initiation, but not the maintenance, of PE gene expression.

Finally, we examined the status of the EPI in *Sox2* null embryos around the time of implantation, because EPI cells are not detected in *Sox2* null embryos postimplantation [5]. In *Sox2* null embryos at E4.25, expression of the EPI marker PECAM1 [14], [47] was undetectable (4/5 *Sox2* null embryos) or reduced (1/5 *Sox2* null embryos), expression of OCT4 was undetectable (1/5 *Sox2* null embryos) or reduced (3/5 *Sox2* null embryos), and expression of NANOG was also undetectable (1/1 *Sox2* null embryos) (Fig. 6G). These observations indicate that although *Sox2* is dispensable for the initiation of EPI gene expression, *Sox2* is required to maintain EPI gene expression. To evaluate the role of *Sox2* in the EPI at later developmental stages, we prolonged preimplantation by inducing diapause. By two to four days of delayed implantation, the number of presumptive EPI cells was reduced in *Sox2* null embryos relative to wild type, while the number of PE cells was largely maintained until the latest time point (Fig. S4D). We conclude that *Sox2* is required for maintaining EPI cell fate at E4.25 and thereafter.

## Discussion

Here we have examined the roles and regulation of SOX2 in the preimplantation embryo, with the goal of deepening our understanding of the origins of pluripotency during development. We showed that SOX2 is a unique, early marker of ICM progenitors, consistent with the reported early enrichment of *Sox2* mRNA in ICM progenitors [14]. However, the significance of this early expression is not obvious, since the cell-autonomous role for *Sox2* in regulating cell fate does not become apparent until late blastocyst stage. It is possible that *Sox2* is initially genetically redundant with other pluripotency factors, such as *Oct4* or *Nanog*. Although phenotypes resulting from disruption of multiple pluripotency genes have not yet been reported in mice, there is evidence of genetic redundancy among zebrafish *Oct/Sox/Nanog* orthologues [48]. Genetic redundancy between these factors is consistent with our observations that *Fgf4* expression is reduced, but not eliminated, in the absence of either *Sox2* or *Oct4* [3]. Thus, in the embryo, OCT4 and SOX2 may promote expression of *Fgf4*, and possibly other targets, synergistically, as has been demonstrated in pluripotent stem cell lines [38], [39].

**Figure 7:**
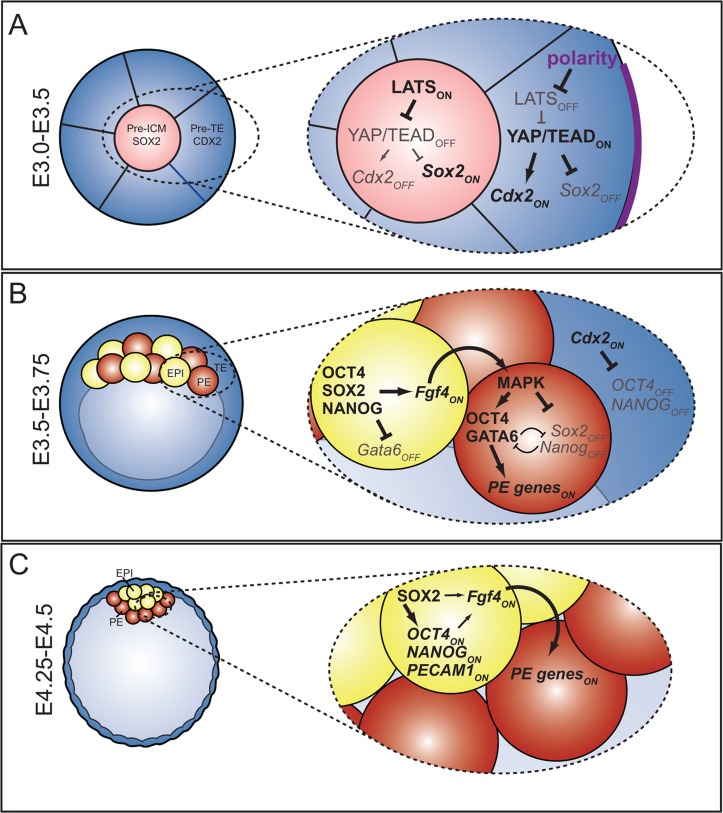
The roles and regulation of SOX2 during blastocyst formation. A) At the 16-cell stage, when ICM progenitors first arise, HIPPO pathway members regulate expression of TE (*Cdx2*) and ICM (*Sox2*) genes in parallel. At this stage, OCT4 and NANOG are still expressed ubiquitously. B) In the blastocyst, *Sox2* expression is restricted to EPI cells by FGFR/MEK signaling. In EPI cells, SOX2 helps promote expression of *Fgf4*, which signals to neighboring cells to induce expression of PE genes. In PE cells, MAPK promotes PE gene expression in an *Oct4*-dependent manner [3], [13], [59], and represses expression of *Sox2* and *Nanog* in PE cells either directly or indirectly. C) In the late blastocyst, SOX2 helps maintain expression of pluripotency genes, and FGF4, or other signals from EPI, maintain expression of PE genes in neighboring cells.

Our evidence suggests that SOX2 and CDX2 are patterned by HIPPO pathway components in parallel (Fig. 7A), but it is not yet clear whether SOX2 and CDX2 are regulated by HIPPO pathway components in the same way. In the TE, expression of CDX2 is activated by a YAP/WWTR1/TEAD complex [24]–[26]. Here we showed that TEAD4 represses expression of SOX2, but we do not yet know if TEAD4 regulates expression of *Sox2* directly or indirectly. In ES cells, the YAP/TEAD complex been shown to bind upstream of *Sox2* to promote its expression [49], arguing that TEAD4 could, in principle, work together with a transcriptional repressor to repress expression of *Sox2* in TE cells. It will be exciting to address this hypothesis in future studies in addition to examining whether position, polarity, and/or contact regulate *Sox2* expression, as has been shown for *Cdx2* [26], [50]–[54]. Interestingly, the HIPPO pathway can be activated in a position-independent manner in blastomeres [53], [55], raising the possibility that multiple upstream inputs could regulate expression of genes such as *Sox2* and *Cdx2* in the embryo. Finally, our observations are also consistent with LATS regulating the activity of an as-yet unidentified transcription factor that promotes expression of SOX2 in ICM progenitors. This hypothesis is also supported by qPCR evidence that *Lats1/2* maintains expression of *Sox2* in the blastocyst [56]. Identification of LATS targets in the preimplantation embryo will therefore provide exciting new inroads into understanding the origins of pluripotency.

Our study provides insight into the regulation of extraembryonic cell types during preimplantation development. In terms of the TE lineage, we showed that SOX2 is not detected in TE cells during preimplantation, nor is it required for their specification. These observations suggest that expression of *Sox2* is activated de novo in the trophoblast lineage postimplantation, where it promotes trophoblast development [5], and raise the possibility that HIPPO pathway components participate in regulation of *Sox2* expression postimplantation as well. Investigation of the mechanisms by which *Sox2* expression becomes activated in the extraembryonic ectoderm is an exciting opportunity to learn about the origins of trophoblast stem (TS) cells, which are derived from extraembryonic ectoderm [57], and dependent on *Sox2* and *Tead4* [15], [26].

While SOX2 does not activate TE gene expression, SOX2 also does not appear to repress TE gene expression in the ICM. This is in contrast to OCT4, which is known to repress expression of TE genes in the ICM and in ES cell lines [3], [7], [10], [58], [59]. Moreover, we have shown that SOX2 is neither expressed nor functional in PE cells at the time when OCT4 represses TE gene expression in PE cells [3]. These observations support the idea that SOX2 and OCT4 have important non-overlapping roles in the embryo and stem cell lines [15], [60], in addition to their widely appreciated overlapping functions. Thus in the ICM, OCT4 may act alone or with partners other than SOX2 to repress transcription of TE genes in EPI cells.

Our analysis led us to explore the genetic regulation of PE specification as well. We have shown that in the ICM, SOX2 becomes expressed in a salt-and-pepper fashion, similar to NANOG. We have also shown that the salt-and-pepper distribution of SOX2 in the ICM depends on FGFR/MEK signaling, but it is not yet clear whether FGFR/MEK signaling regulates SOX2 expression directly, or whether FGFR/MEK signaling maintains cell fate, which in turn regulates SOX2 expression. Alternatively, NANOG or GATA6 could help regulate SOX2 expression in the ICM. While the expression pattern of SOX2 in *Nanog* null embryos has not yet been reported, in *Gata6* null embryos, SOX2 is expressed in all ICM cells [61], suggesting that GATA6 helps mediate FGFR/MEK signaling to repress SOX2 in the E3.75 ICM (Fig. 7B). Further studies of SOX2 in *Gata6* and *Nanog* null embryos, with and without manipulations to the FGFR/MEK signaling pathway will help clarify the direct and indirect mechanisms regulating *Sox2* expression in the ICM.

We also showed that SOX2 helps to maintain the appropriate level of FGF4 that is essential for timely creation of the hypoblast layer. Curiously, *Sox2* null embryos do not completely phenocopy *Fgf4* null embryos, since NANOG was not upregulated in *Sox2* null embryos as it is in *Fgf4* null embryos [35]. We hypothesize that the intermediate level of *Fgf4* in *Sox2* null embryos are sufficient to repress NANOG, as we have shown for *Oct4* null embryos [3]. In addition, we did not observe reduced total cell number in *Sox2* null embryos as has been observed in *Fgf4* null embryos [35], arguing that a moderate level of FGF4 can maintain cell proliferation during preimplantation. Finally, the moderate level of FGF4 produced by *Sox2* null embryos is eventually able to restore expression of PE genes (Fig 7C), which does not occur in embryos completely lacking *Fgf4* [35], [37], or downstream signaling components [4]. Interestingly, the timing of PE gene expression has also been shown to be sensitive to dose of *Gata6* [61], suggesting *Fgf4* may be regulated by GATA6 as well. By contrast, PE gene expression is not eventually restored in *Oct4* null or in *Nanog* null embryos [3], [9]. In light of evidence that *Oct4* is not required for later expression of PE genes [59], our observations predict that *Fgf4* levels, or levels of an as-yet unidentified, later-acting signal essential for maintaining PE gene expression, may be more rapidly and/or dramatically lost in *Oct4* and possibly *Nanog* null embryos, than in *Sox2* null embryos.

Our observation that SOX2 is one of the earliest known unique markers of ICM progenitors is supported by the observation that SOX2 is also one of the first pluripotency genes to localize to ICM progenitors in the morula in other mammals [62]. While *Sox2* is not initially required for expression of pluripotency genes in the mouse, *Sox2* eventually does promote expression of pluripotency genes in the ICM, as in ES cells. Thus, in the ICM, the role of *Sox2* appears to be to maintain, and not to initiate, pluripotency (Fig. 7C). This idea is consistent with observations that pluripotency genes are initially normal in *Oct4* and *Nanog* null embryos [3], [12], [33]. The idea of a later role for *Sox2* in maintaining expression of pluripotency genes in the ICM is also consistent with evidence that both the genetic regulation and the transcriptional profile of ES cells are more similar to late than to early EPI cells [7], [63]. Recent studies have shown that HIPPO and IL6/JAK/LIF/STAT3 pathways also maintain expression of pluripotency genes in the ICM around implantation stage [50]–[52], [64]. Discovering the mechanisms of crosstalk between pluripotency pathway members at the implantation stage and shortly thereafter will therefore provide exciting new insight into the origins of pluripotent stem cells.

## Materials and Methods

### Mouse strains and genotyping

All animal research was conducted in accordance with the guidelines of the University of California Santa Cruz Institutional Animal Care and Use Committee or by RIKEN CDB and Kumamoto University. The following alleles or transgenes were used in this study: *Sox2^tm1.1Lan^* [27], *Tg(Zp3-cre)93Knw* [28], *Tead4^tm1Hssk^* [24], and *Cdx2^tm1.1Aral^* [21]. Mice carrying the Sox2 null allele (*Sox2^del+^*) were generated by crossing mice carrying *Sox2^tm1.1Lan^* with *129-Alpl^tm1(cre)Nagy^* [65].

### Embryo collection and manipulation

As described previously [3], [21], mice were maintained on a 12-hour light/dark cycle. Embryos were collected from timed natural matings by flushing dissected oviducts or uteri with M2 medium. Cultured embryos were cultured in KSOM (Millipore) alone, or KSOM with a final concentration of 1 µg/ml recombinant human FGF4 (R&D Systems) and 1 U/mL Heparin (Sigma), or 100 nM PD173074 and 500 nM PD0325901 (Stemgent) at 37°C and 6% CO_2_. Microinjection of mRNA was performed as described [26], [50]. To delay implantation, diapause was induced as previously described [32], [66], [67].

### Immunofluorescence and confocal microscopy

Embryos were fixed, stained, imaged, and recovered for genotyping as previously described [68]. Primary and secondary antibody sources were described previously [3], [50], and also included rabbit anti-EOMES (Abcam), rabbit anti-DAB2 (Santa Cruz Biotech), rabbit anti-LAMA1 (Sigma), and rat anti-PECAM1 (BD Biosciences).

### Chimeras

Chimeras were performed using *Sox2* MZ null embryos as previously described [3]. Chimeras were subsequently genotyped by PCR using primers that distinguished wild-type, floxed, and deleted *Sox2* alleles [27].

### RNA isolation and cDNA preparation

RNA isolation and single blastocyst qPCR was performed as previously described [21]. qPCR primers included *Sox2* (GCGGAGTGGAAACTTTTGTCC and CGGGAAGCGTGTACTTATCCTT), *Fgf4* (AGCAGGGGCAAGCTCTTC and GGGTACGCGTAGGATTCG), *Oct4* (AGCTGCTGAAGCAGAAGAGG and AGATGGTGGTCTGGCTGAAC), and *Actb* (CTGAACCCTAAGGCCAACC and CCAGAGGCATACAGGGACAG).

## Supporting Information

Figure S1 Details of SOX2 expression pattern in the early embryo. A) In morulae lacking *Sox2*, the anti-SOX2 antibody non-specifically detects a cytoplasmic pattern in TE cells, while the signal is specific to ICM at the blastocyst stage. B) SOX2 is detected in an increasing proportion of inside/ICM cells during formation of the blastocyst, with nearly 100% of ICM cells expressing SOX2 by E3.5. C) SOX2 does not colocalize with CDX2 in TE cells of the blastocyst, although SOX2 colocalizes with rare inside cells that express CDX2 (arrowhead, n = 3/13 embryos), consistent with our prior observations that CDX2 is detected in rare inside cells at this stage [68]. Bar  = 20 µm.
(EPS)

Figure S2 M Sox2 is not detectable in early embryos, and SOX2 does not repress TE fate in the ICM. A) M SOX2 protein is not detectable in wild type zygotes (*Sox2* M null evaluated as a negative control), M *Sox2* mRNA is not detectable (*) in individual wild type 2-cell embryos by qPCR (*Oct4* evaluated as a positive control), and M SOX2 is not detectable in nuclei of *Sox2* Z null embryos at the 16-cell stage (non-mutant as positive control for SOX2 signal). B) At E4.5, neither CDX2 nor EOMES are detected in the ICM of *Sox2* null embryos. Bar  = 20 µm.
(EPS)

Figure S3 Z and not M *Sox2* acts upstream, and not downstream of *Fgf4*. A) qPCR analysis of single blastocysts at E3.75 shows that M *Sox2* is not required for *Fgf4* expression. B) Treatment of control (*Sox2* M null or nonmutant) or *Sox2* null (MZ or Z null) embryos for 42 h starting at E2.75 leads to complete upregulation of SOX17 and downregulation of NANOG throughout the ICM. C) Quantification of embryos shown in panel B (avg. no. cells per ICM: untreated nonmutant 17.9+/−4.3; untreated *Sox2* null: 8.0+/−1.4; treated nonmutant: 17.1+/−5.6; treated *Sox2* null: 18.0+/−4.1). Bar  = 20 µm, p-value calculated by t-test; n.s.  = p>0.05; ANOVA performed for panel C.
(EPS)

Figure S4 Evaluation of PE quality in late blastocysts. A) At E4.25, the average proportion of ICM cells in which GATA6 is detected is equivalent between control and *Sox2* null blastocysts. B) Average ICM cell number for time points examined in Fig. 6B. C) Expression of DAB2 and localization of PE cells is rescued by wild type ES cells in *Sox2* null blastocysts at the equivalent of E4.25. D) Expression of DAB2 is eventually rescued in *Sox2* null implantation-delayed blastocysts. The number of EPI cells is significantly reduced in *Sox2* null implantation-delayed blastocysts, relative to wild type. By contrast, the number of PE cells is not significantly reduced in *Sox2* null implantation-delayed blastocysts, relative to wild type, until the last time point examined. EPI and PE were defined based on SOX17 expression, since other EPI markers are not detectable in *Sox2* null blastocysts at this stage. Bar  = 20 µm, p-value calculated by t-test, n.s.  = p>0.05.
(EPS)

Table S1 Cell numbers detected in wild type embryos harvested at the indicated times (E3.0–E4.5).
(DOCX)
