# Supplementary material for: HIPPO Pathway Members Restrict SOX2 to the Inner Cell Mass Where It Promotes ICM Fates in the Mouse Blastocyst
Source: PLoS Genet. 2014 Oct 23;10(10):e1004618. doi: 10.1371/journal.pgen.1004618 (PMC4207610; doi:10.1371/journal.pgen.1004618)
Supplement: Table S1 — Cell numbers detected in wild type embryos harvested at the indicated times (E3.0–E4.5). (DOCX) [file pgen.1004618.s005.docx]

**Table S1. Cell numbers detected in wild type embryos harvested at the indicated times (E3.0-E4.5).**

|  | **Cell number (range)** | **Cell number**  **(average)** | **Standard deviation from average** | **Number of embryos** |
| --- | --- | --- | --- | --- |
| E3.0 | 13-23 | 16 | 3 | 10 |
| E3.25 | 24-42 | 31 | 4 | 4 |
| E3.5 | 43-63 | 58 | 4 | 19 |
| E3.75 | 64-80 | 72 | 5 | 21 |
| E4.0 | 81-100 | 89 | 5 | 12 |
| E4.25 | 101-135 | 113 | 10 | 6 |
| E4.5 | 130-180 | 161 | 13 | 5 |
